# Supplementary material for: Causal Relationship between Aging and Anorexia Nervosa: A White-Matter-Microstructure-Mediated Mendelian Randomization Analysis
Source: Biomedicines. 2024 Aug 16;12(8):1874. doi: 10.3390/biomedicines12081874 (PMC11351342; doi:10.3390/biomedicines12081874)
Supplement: Supplementary file 1 [file biomedicines-12-01874-s001.zip › Supplementary Figure/Figure S4/Singe SNP plot of MR analysis for TL to ubm-b-1929.pdf]

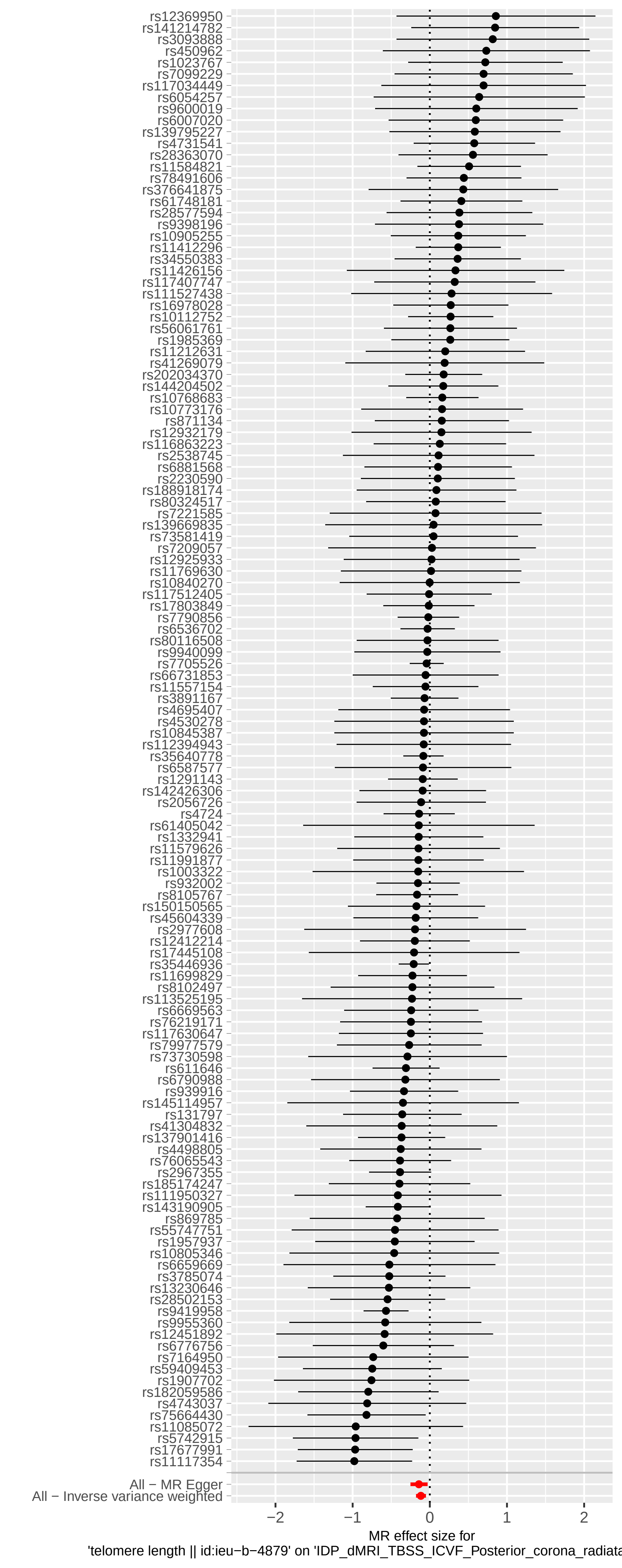
rs12369950
rs141214782
rs3093888
rs450962
rs1023767
rs7099229
rs117034449
rs6054257
rs9600019
rs6007020
rs139795227
rs4731541
rs28363070
rs11584821
rs78491606
rs376641875
rs61748181
rs28577594
rs9398196
rs10905255
rs11412296
rs34550383
rs11426156
rs117407747
rs111527438
rs16978028
rs10112752
rs56061761
rs1985369
rs11212631
rs41269079
rs202034370
rs144204502
rs10768683
rs10773176
rs871134
rs12932179
rs116863223
rs2538745
rs6881568
rs2230590
rs188918174
rs80324517
rs7221585
rs139669835
rs73581419
rs7209057
rs12925933
rs11769630
rs10840270
rs117512405
rs17803849
rs7790856
rs6536702
rs80116508
rs9940099
rs7705526
rs66731853
rs11557154
rs3891167
rs4695407
rs4530278
rs10845387
rs112394943
rs35640778
rs6587577
rs1291143
rs142426306
rs2056726
rs4724
rs61405042
rs1332941
rs11579626
rs11991877
rs1003322
rs932002
rs8105767
rs150150565
rs45604339
rs2977608
rs12412214
rs17445108
rs35446936
rs11699829
rs8102497
rs113525195
rs6669563
rs76219171
rs117630647
rs79977579
rs73730598
rs611646
rs6790988
rs939916
rs145114957
rs131797
rs41304832
rs137901416
rs4498805
rs76065543
rs2967355
rs185174247
rs111950327
rs143190905
rs869785
rs55747751
rs1957937
rs10805346
rs6659669
rs3785074
rs13230646
rs28502153
rs9419958
rs9955360
rs12451892
rs6776756
rs7164950
rs59409453
rs1907702
rs182059586
rs4743037
rs75664430
rs11085072
rs5742915
rs17677991
rs11117354
All − MR Egger
All − Inverse variance weighted
−2
−1
0
1
2
MR effect size for
'telomere length || id:ieu−b−4879' on 'IDP_dMRI_TBSS_ICVF_Posterior_corona_radiata
